# Novel insights into peptide amidation and amidating activity in the human circulation

**DOI:** 10.1038/s41598-021-95305-y

**Published:** 2021-08-04

**Authors:** Paul Kaufmann, Andreas Bergmann, Olle Melander

**Affiliations:** 1 PAM Theragnostics GmbH, 16761 Hennigsdorf, Germany; 2 Sphingotec GmbH, 16761 Hennigsdorf, Germany; 3 Department of Clinical Sciences Malmö, Lund University, 205 02 Malmö, Sweden; 4 Department of Emergency and Internal Medicine, Skåne University Hospital, Malmö, Sweden

**Keywords:** Enzymes, Peptide hormones, Predictive markers, Heart failure, Hypertension

## Abstract

C-terminal α-amidation is the final and essential step in the biosynthesis of several peptide hormones. Peptidylglycine α-amidating monooxygenase (PAM) is the only known enzyme to catalyse this reaction. PAM amidating activity (AMA) is known to be present in human circulation, but its physiological role and significance as a clinical biomarker remains unclear. We developed a PAM-specific amidation assay that utilizes the naturally occurring substrate Adrenomedullin-Gly (ADM-Gly, 1–53). Using our amidation assay we quantified serum amidating activities in a large population-based cohort of more than 4900 individuals. A correlation of serum amidating activity with several clinical parameters including high blood pressure was observed. Increasing PAM-AMA was an independent predictor of hard outcomes related to hemodynamic stress such as cardiovascular mortality, atrial fibrillation and heart failure during long-term follow-up (8.8 ± 2.5 years). Moreover, results from an animal study in rats utilizing recombinant human PAM provide novel insights into the physiological role of circulating PAM and show its potential significance in circulating peptide amidation.

## Introduction

Biologically active peptide hormones fulfill functions as neurotransmitters and paracrine and endocrine signaling agents in a large number of organisms. Despite great diversity in size and sequence, signaling peptides mostly share a common biosynthetic pathway: they originate from large, inactive precursors and undergo several co- and posttranslational modifications, including cleavage of signal peptides, endoproteolytic cleavage of precursor propeptides by specific endopeptidases, formations of disulfide bonds and glycosylation^1^. More than seventy neural and endocrine peptides require a C-terminal α-amide group to gain biological activity^2,3^. C terminal α-amidation represents the final and essential step in peptide hormone biosynthesis. This reaction is exclusively catalyzed by peptidylglycine α-amidating monooxygenase (PAM)^4–6^. PAM is a bifunctional enzyme consisting of two catalytical subunits, peptidylglycine α-hydroxylating monooxygenase (PHM) and peptidyl-α-hydroxyglycine α-amidating lyase (PAL). α-amidation is therefore a two-step reaction involving stereospecific hydroxylation of the α-carbon in the free C-terminal glycine of a peptidylglycine substrate. This first partial reaction is catalyzed by the Cu and Ascorbate dependent PHM (EC 1.14.17.3)^7–9^. The second part of the amidation involves C–N bond cleavage of the α-hydroxylated product and release of glyoxylate by the Zn-dependent PAL (EC 4.3.2.5). The transfer of the intermediate peptidyl-α-hydroxyglycine from PHM to PAL is not completely understood. Recent data suggest a release of the intermediate reaction product into solution by PHM rather than a direct shuttling mechanism to PAL^10^.

Besides several others, three main isoforms of PAM are known. While isoforms 1 and 2 are both membrane bound within the lumen of secretory vesicles^2,11^ of the constitutive and regulated secretion pathway, isoform 3 is soluble and is secreted^2,12^. PAM activities were analyzed in several human body fluids including CSF, serum and plasma^13–15^. Initial proof of circulating amidating activity by Wand et al. revealed some variations in certain disease states^13,15^. Besides the work of Gaier et al., where PHM and PAL activities in 120 frail men were analyzed, serum or plasma PAM activities have not been studied in any large human population-based cohort so far^16^. Furthermore, no attempts were made to quantify the concentration of circulating PAM and until today the physiological role of PAM in circulation is not understood^16^.

On part of PAM-substrates, several reports described circulating peptidylglycines and their amidated counterparts. Circulating glycine-extended forms were described for several peptides undergoing amidation^17–19^. Examples of the best studied glycine-extended peptides are gastrin, cholecystokinin (CCK) and adrenomedullin (ADM)^17^. The major circulating form of ADM is ADM-Gly^19^. No experimental proof with respect to amidation of circulating peptidylglycines by circulating PAM was reported in the literature to date. Since peptidylglycine precursors are co-secreted with their amidated counterparts and peptidylglycines appear to have no biological function^17,18^, an intriguing question is whether circulating PAM is capable of amidation of circulating peptidylglycine substrates or whether circulating PAM is a waste-product of the secretory pathway. Previous animal studies stated a connection of administration or deprivation of ascorbate, known to influence PAM activity^2^, with changes in levels of amidated peptides^20–22^. In humans, the importance of ascorbate as a cofactor for PAM was mentioned in connection to the beneficial effects of vitamin C administration and sepsis outcome. It was speculated that administration of vitamin C could have stimulatory effects on PAM, thereby contributing to amidation of peptides known to be depleted in sepsis, such as vasopressin^23–26^. Furthermore, several Type 2 Diabetes Risk alleles were identified in *PAM* encoding for PAM proteins with impaired activity^27–29^. The mechanism suggested by Thomsen et al., proposes impaired Chromogranin A amidation resulting in adverse insulin granule packaging and insulin secretion^29^. Besides its indisputable catalytical roles, PAM was shown lately to have non-catalytic but structural giving roles in the assembly of atrial myocyte secretory granules, thereby impacting secretion of atrial natriuretic peptide (ANP)^30,31^.

In this work, we developed an activity assay for PAM using human ADM-Gly as substrate and addressed three main questions: (1) What is the distribution and relation to clinical characteristics and hard outcomes of circulating PAM activity in a large human population-based cohort of more than 4900 individuals. (2) Is circulating PAM capable of α-amidation of circulating ADM-Gly in an in vivo rat model? (3) Does ascorbate affect circulating PAM activity with respect to ADM-Gly to bio-ADM conversion in an in vivo rat model?

## Results

### Amidating activity (AMA) assay

#### Optimal cofactor concentration

**Figure 1:**
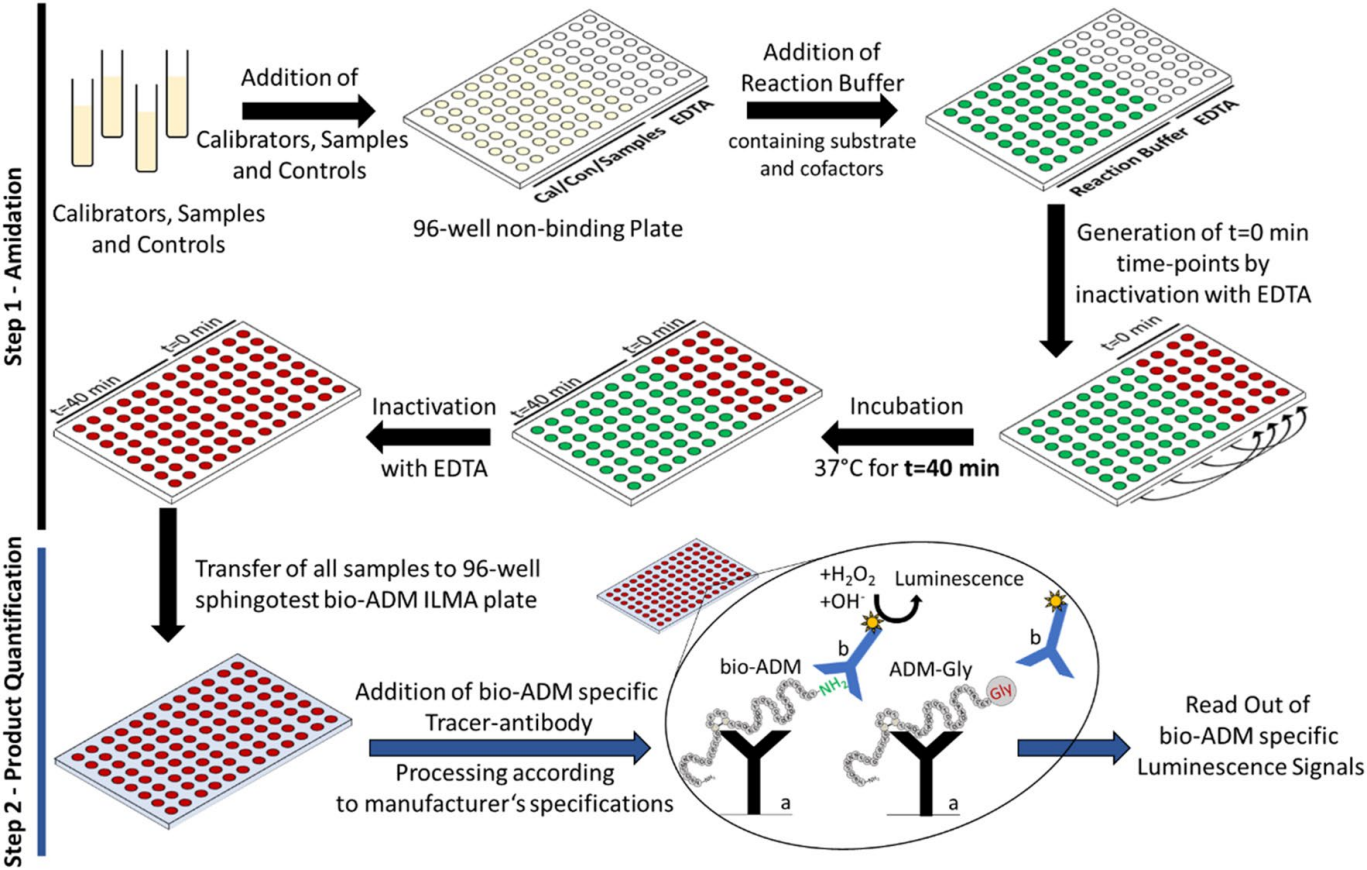
Schematic representation of the AMA Assay. Step 1—amidation: calibrators, controls and samples are dispensed in double determination as indicated (yellow) into a non-binding 96-well plate. The reaction is started by addition of reaction buffer to calibrators, controls and samples (green) and a t = 0 min time-point is generated immediately by transferring equal volumes of each double determination into EDTA prefilled wells (red). The generated t = 0 min time-points are single determinations. After incubation at 37 °C for 40 min an inactivation of remaining samples with EDTA is performed. Step 2—product quantification: following 37 °C incubation and inactivation, all samples are transferred into the sphingotest bio-ADM Assay 96-well immunoluminometric assay (ILMA) plate. The bio-ADM assay is processed according to manufacturer’s specifications. Both, ADM-Gly and bio-ADM are bound by the solid-phase antibody (a). The MACN labelled tracer-antibody (b) specifically recognizes the c-terminally amidated bio-ADM and does not react with ADM-Gly. Following incubation, unbound tracer-antibody is washed away and bio-ADM specific luminescence signals are detected using a standard plate luminometer. The figure was created with Microsoft PowerPoint 2016, version 2105.

We developed and validated a two-step assay that uses human ADM-Gly as substrate to specifically detect PAM amidating activity in human plasma and serum. In the first step bio-ADM is produced from 1–53 ADM-Gly, and in the second step generated bio-ADM is quantified with the sphingotest bio-ADM assay (Fig. 1, Fig. S1)^32^. The degradation of bio-ADM, the half-life of which is estimated to approximately 20 min in serum^33^, was prevented with an N-terminal anti-ADM antibody^32^ in combination with amastatin and leupeptin. As shown in Fig. S2, after 60 min of incubation at 37 °C, 82% of ADM-Gly and 92% of bio-ADM remained stable in serum. Since copper and ascorbate concentrations varied in several reports^13,14,16,34^, we analyzed the optimal cofactor concentrations for the conversion of ADM-Gly to bio-ADM by native human PAM. The optimal concentrations were 2 mM and 5 µM for ascorbate and Cu^2+^, respectively (Fig. S3), and were used as standards for all PAM-AMA assays unless specified otherwise.

#### Analytical assay characteristics

**Figure 2:**
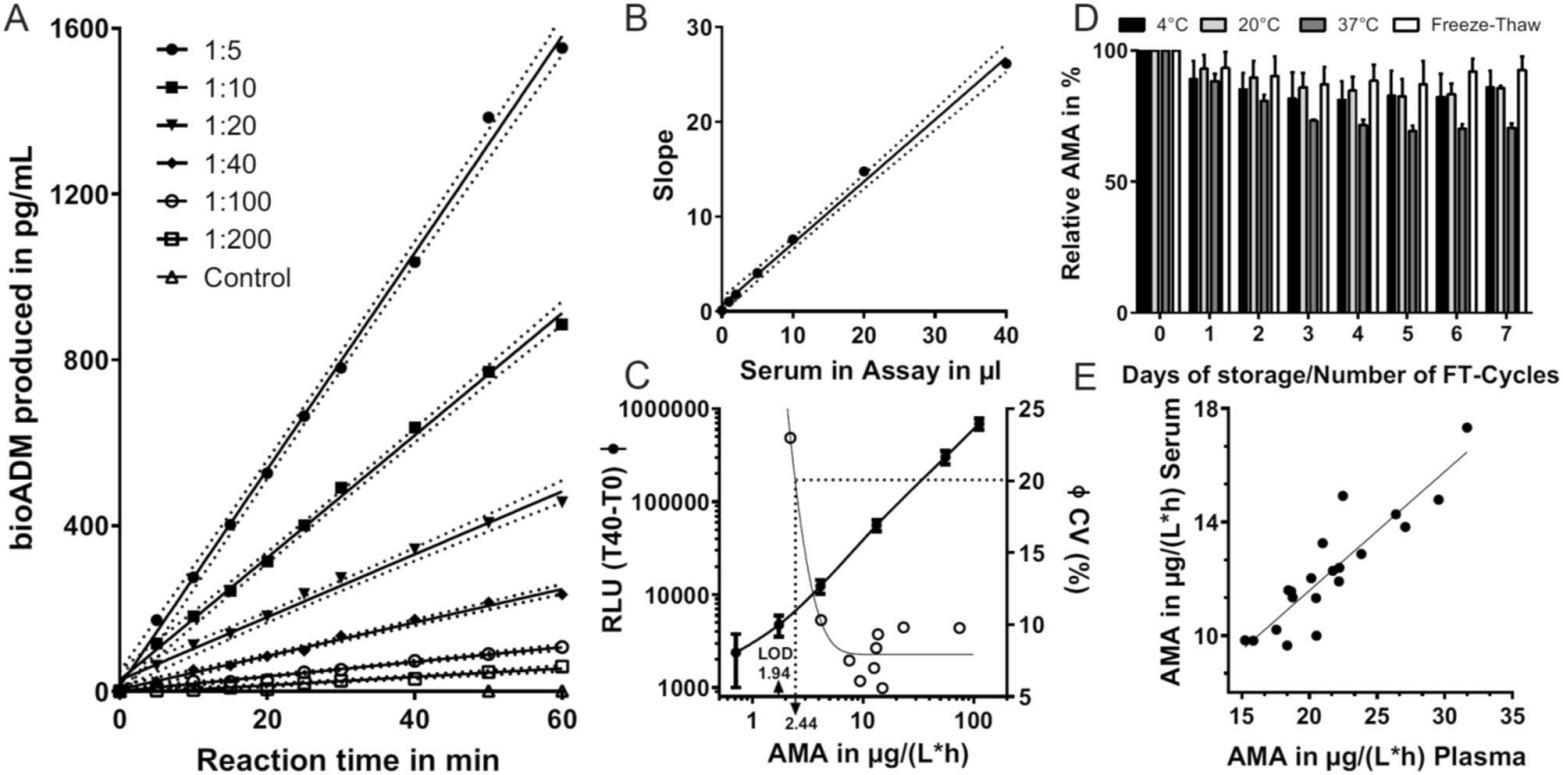
AMA Assay characteristics and analyte stability. (**A**) Time-dependent product formation linearity with varying serum dilutions as source of human native PAM in assay. Product formation linearity of each dilution was verified with Runs Test. (**B**) Slopes from each serum dilution (**A**) were plotted against the volume of serum used in the assay. The 95% confidence interval is shown as dotted lines in (**A**) and (**B**). (**C**) Representative dose–response curve of enzymatic calibrator (bold line, closed circles) and interassay precision profile (empty circles) for the determination of PAM-AMA. Each point represents a mean of 16 measurements. (**D**) Stability analysis of native human PAM-AMA in serum as source of PAM. Activity of treated material was normalized to activity of untreated material that was set as 100%. (**E**) Spearman-correlation of PAM-AMA from matched serum and Li-heparin plasma samples (r = 0.89; p < 0.0001; n = 22).

Time dependent linearity of bio-ADM formation by native human PAM was observed in all serum dilutions within 60 min and confirmed via Runs test (Fig. 2A). Bio-ADM formation by native human PAM was linear in the range of 1–40 µL of sample matrix used in the assay (Fig. 2B). Based on these data tenfold dilutions of sample matrix (e.g. plasma or serum) were used in further assays corresponding to 20 µL of serum or plasma per reaction.

The assay was calibrated using a dilution series of recombinant human PAM with pre-defined amidating activities. The LOQ and LOD of the AMA assay were 2.44 AMA Units and 1.94 AMA Units, respectively, as determined from the typical dose response curve along with the inter assay precision of the PAM-AMA assay (Fig. 2C, Table 1).

**Table 1:** Performance characteristics of enzymatically calibrated PAM-AMA Assay.

Analytical performance characteristics of the AMA assay	
Calibrator range	0.712–121.865 AMA units (0–4000 ng/mL)
Intra-assay CV	3–6%
Limit of detection	1.94 AMA units (42.4 ng/mL)
Limit of quantification	2.44 AMA units (60.4 ng/mL)
Accuracy (recovery of added PAM to serum)	Average (range) % recovery from expected = 108% (103–116%)
Linearity (mixing studies) 12 ×	Range: 3.2–16.5 AMA units Average (range) % recovery from expected = 91% (82–101.6%)
Dilution linearity (10–100 × in assay)	Range: 0–16.5 AMA units Average (range) % recovery from expected = 98% (91–103%)
Variation of substrate concentration CV	Range: 10–40 ng/mL; CV: 1.2–2.4%

Further characterization, performance and stability analyses of the PAM-AMA assay are summarized in Table 1. To assess the accuracy of the assay, serum spiked with recombinant human PAM was diluted in human serum of known AMA. The AMA recovery was in the range of 103–116% and fulfilled the acceptance criteria for a deviation from expected concentration of ± 20% (Table 1). To assess the linearity of the assay, stepwise dilution studies of native serum and mixing studies were performed. Both approaches resulted in an AMA recovery from the expected value within a range of 82–103% (Table 1). Further we analyzed the effect of varying substrate concentrations on PAM-AMA fitted from recombinant calibrator material. Within the substrate range of 10–40 ng/mL AMA of the sample correlated well with the amidating activity of the calibrator (Fig. S4).

The ex-vivo AMA stability was assessed in three human serum samples. Samples retained 92%, 90% and 85% amidating activity after storage at RT for 24 h, 2 and 7 days, respectively (Fig. 2D). 86% of activity retained after 7 days, when serum was kept at 4 °C (Fig. 2D). Upon storage at 37 °C 89% of initial activity was detectable after 24 h, while after 7 days AMA decreased to 70%. After 7 freeze–thaw cycles of human serum 92% of amidating activity remained in the samples (Fig. 2D).

To ensure the accurate comparison between AMA´s measured in Li-heparin and serum, we analysed 22 matched serum and Li-heparin pairs (Fig. 2E). Matched activities showed a correlation of r = 0.89 (p < 0.0001). Serum activities were, therefore, considered comparable to Li-heparin activities when adjusted by the factor of 1.6.

### AMA distribution in a human population

**Figure 3:**
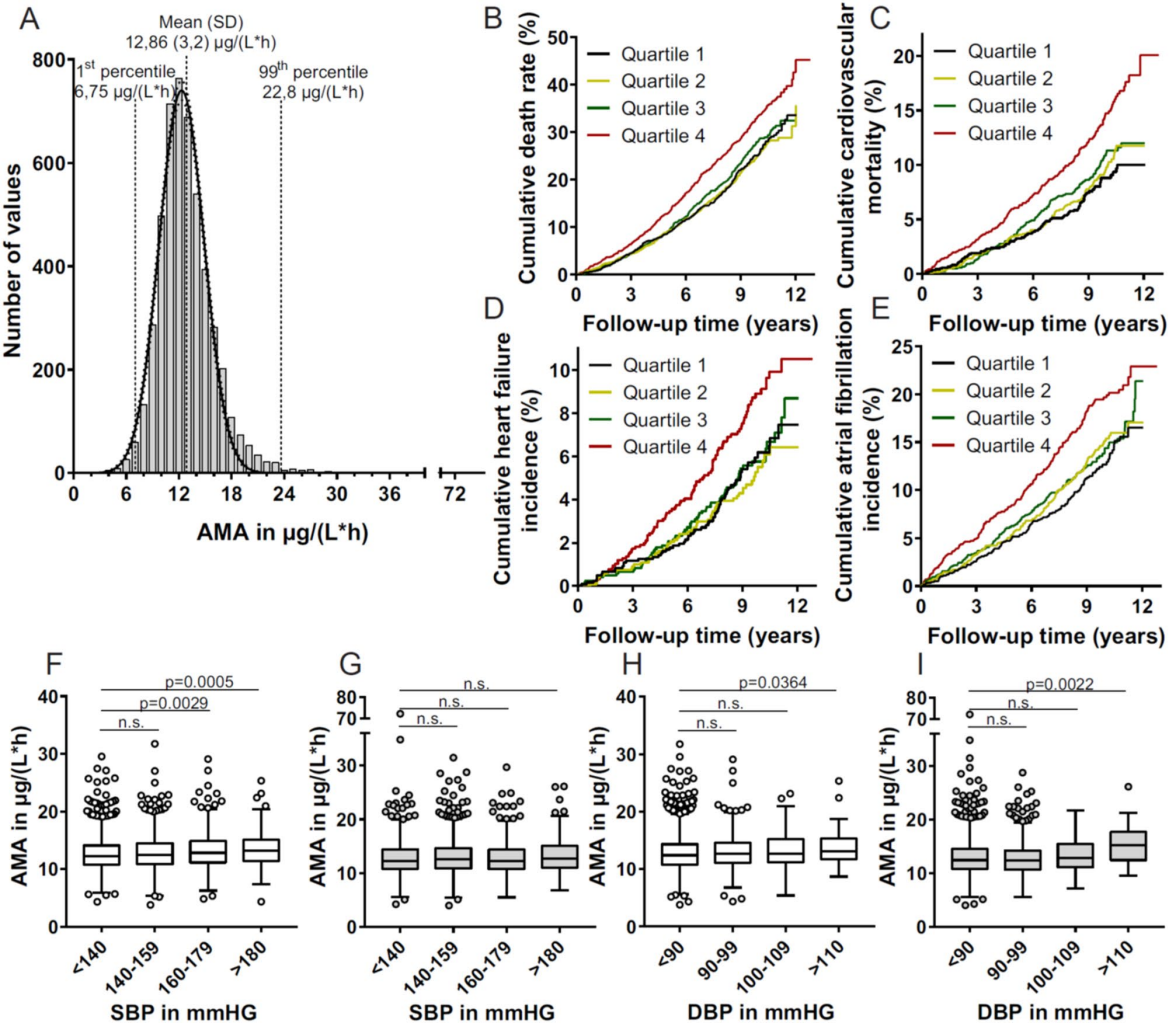
PAM-AMA analysis in n = 4942 individuals from the population based MPP study cohort. (**A**) Frequency distribution of PAM-AMA. The data did not pass the D’Angostino–Pearson omnibus test for normal distribution; a normal distribution curve (solid line) was fitted into the frequency distribution of the cohort. The 95% CI is shown as dotted lines. Kaplan Meier analysis for all-cause mortality risk (**B**), cardiovascular mortality risk (**C**), heart failure risk (**D**) and atrial fibrillation risk (**E**) in relation to serum AMA levels. Subjects analyzed in (**C**) and (**D**) were free of heart failure or atrial fibrillation at baseline. Over the follow-up time of 12.8 years the cumulative rates of each indication differed between the highest quartile of AMA (Q4) and the lowest Quartile of AMA (Q1). Quantitative analyses are summarized in Table 4 (**B**,**C**) and Table 5 (**D**,**E**). PAM-AMA in subjects from the MPP-cohort was categorized according to systolic- (**F**,**G**) and diastolic blood pressure (**H**,**I**). The population was divided in subject not receiving antihypertensive treatment (**F**,**H**) and in subjects receiving antihypertensive treatment (**G**,**I**). Difference in PAM-AMA was analyzed via one-way ANOVA (Dunn’s correction). Significance of differences is shown in each panel, *n.s.* not significant.

To investigate the distribution of PAM in a large number of individuals, we determined PAM-AMA in a random sub-cohort of the Malmoe Preventive Project (MPP)^35^. Figure 3A shows a frequency distribution of PAM-AMA in 4942 individuals with a mean activity of 12.86 (± 3.2) AMA units. The 99th percentile was 22.8 AMA units. The frequency distribution showed a gauss-like behaviour but did not pass the D’Agostino Pearson omnibus test for normal distribution.

Baseline characteristics of the study population after stratification for quartiles (Q) of AMA are shown in Table 2. We observed slight but significant differences between quartiles of AMA for age, smoking prevalence, systolic blood pressure (SBP), diastolic blood pressure (DBP), diabetes mellitus prevalence and glucose level. Spearman correlations of AMA with clinical parameters within the population are summarized in Table 3. While no significant differences of AMA were observed between females and males (Fig. S5A), AMA was elevated in smokers when compared to non-smokers (p < 0.0001) (Fig. S5B). We observed significant positive correlations of serum AMA with age, SBP, DBP and HDL (Fig. S6A–D). A negative correlation of AMA was observed with the glucose level in the total population (Table 3, Fig. S6E). Interestingly, AMA was lower in subjects with prevalent diabetes and showed a significant negative correlation with glucose level in subjects with prevalent diabetes (Fig. S7A,B), while no correlation was found in subjects free of diabetes (Fig. S7C, Table 3). Further, AMA correlated with bio-ADM (r = 0.064, p < 0.0001) and Mid-regional proadrenomedullin (MR-proADM) (r = 0.11, p < 0.0001) levels (Fig. S8A,B).

**Table 2:** Baseline clinical characteristics according to quartile (Q) of AMA at baseline of subjects analysed.

	Q1	Q2	Q3	Q4	p
	(n = 1235)	(n = 1236)	(n = 1236)	(n = 1235)	
AMA in µg/(L*h) (SD)	9.416 (1.21)	11.66 (0.46)	13.39 (0.57)	17 (3)	N/A
AMA range	3.8–10.86	10.86–12.47	12.47–14.47	14.47–72.15	
Age in years (SD)	68.97 (6.18)	69.16 (6.28)	69.34 (6.38)	70.45 (6.07)	< 0.0001
Gender, n (%) male	870 (70.4)	851 (68.9)	862 (69.8)	812 (65.8)	n.s.
Current smoking, n (%)	188 (15.2)	217 (17.6)	255 (20.6)	287 (23.2)	< 0.0001
Systolic blood pressure in mmHg (SD)	144 (19.77)	145.1 (19.83)	144.8 (20.33)	147.6 (21.34)	< 0.0002
Diastolic blood pressure mmHg (SD)	82.83 (10.12)	84.04 (10.83)	83.12 (10.61)	84.45 (11.51)	0.0041
Antihypertensive medication, n (%)	491 (39.8)	478 (38.7)	458 (37.1)	491 (39.8)	n.s.
Diabetes mellitus, n (%)	166 (13.4)	127 (10.3)	113 (9.1)	127 (10.3)	0.0043
Glucose in mmol/L (SD)	6.024 (1.95)	5.78 (1.21)	5.794 (1.37)	5.753 (1.28)	0.0299
LDL in mmol/L (SD)	3.559 (0.97)	3.646 (1.0)	3.596 (1.0)	3.596 (1.0)	n.s.
HDL in mmol/L (SD)	1.361 (0.38)	1.385 (0.41)	1.387 (0.42)	1.383 (0.41)	n.s.
BMI in kg/m^2^ (SD)	26.94 (3.93)	27.11 (4.34)	27.06 (4.23)	27.12 (4.35)	n.s.

*N/A* not applicable, *n.s.* not significant.

**Table 3:** PAM-AMA spearman rank correlations in the study cohort.

Correlation of AMA to	Spearman r	Number of pairs	p-value
Age	0.0909	4942	< 0.0001
SBP	0.0535	4930	0.0002
DBP	0.0435	4930	0.0022
HDL	0.0338	4937	0.0177
Glucose (total population)	− 0.0389	4942	0.0062
Glucose (prevalent diabetes)	− 0.1132	533	0.0089
Glucose (free of diabetes)	− 0.0157	4409	n.s.

*n.s.* not significant.

During an average follow-up time of 8.8 ± 2.5 years 1361 subjects died. The number of deaths increased with higher quartiles of AMA (Table 4). The highest number of deaths adhered to Q4 (n = 418, Table 4, top). Multivariate adjusted analyses, including adjustment for all baseline PAM correlates and cardiovascular risk factors showed a significantly increased relative risk (26.9%) of all-cause-mortality in the highest versus lowest AMA Quartile (Table 4, top). A total of 480 cv-death events occurred during follow-up with 166 events in Q4 of AMA. Significant elevation of cv mortality risk (55.6%) was observed in Q4 versus Q1 (Table 4, bottom). Both, total- and cv-mortality risk prediction using AMA were independent of common cv-death risk factors. Since mortality appears to be driven by cardiovascular events, we analysed incidence of events associated with hemodynamic stress with respect to AMA. Similar as to cv-mortality, highest versus lowest quartile of AMA was significantly associated with increased risk of incident heart failure (42.8%) and incident atrial fibrillation (55.6%) independently of baseline PAM correlates and cardiovascular risk factors (Table 5 top and bottom, respectively). Prevalent cases were excluded in the analysis of incident AF or HF. Separate analysis of prevalent AF and HF revealed significant elevation of AMA in both conditions (Fig. S9A,B). Kaplan Meier plots by quartiles of AMA of total and cv-mortality, incident AF and HF during follow-up are shown in Fig. 3B–E, respectively. Additionally, we categorized the total population (according to^36^) with respect to blood pressure considering antihypertensive treatment (AHT). In subjects with elevated SBP (grade-2 and garde-3 hypertensives) and with elevated DBP (grade-3 hypertensives) not receiving AHT, AMA was found to be significantly elevated when compared to normotensive subjects (Fig. 3F,H). Subjects who received AHT did not show significant increases in AMA with respect to SBP (Fig. 3G), while DBP in grade-3 hypertensives remained elevated (Fig. 3I).

**Table 4:** Population quartile of AMA in relation to all-cause mortality and cardiovascular mortality.

	Q1	Q2	Q3	Q4
	(n = 1235)	(n = 1236)	(n = 1236)	(n = 1235)
AMA in µg/(L*h) (SD)	9.416 (1.21)	11.66 (0.46)	13.39 (0.57)	17 (3)
AMA range	3.8–10.86	10.86–12.47	12.47–14.47	14.47–72.15
**All-cause mortality**				
Number of events	310	302	331	418
Hazard ratio (95% CI)	(ref)	0.994 (0.847–1.166)^n.s.^	1.031 (0.882–1.205)^n.s.^	1.269 (1.094–1.473)***
**Cardiovascular mortality**				
Number of events	94	106	114	166
Hazard ratio (95% CI)	(ref)	1.172 (0.887–1.548)^n.s.^	1.169 (0.888–1.540)^n.s.^	1.632 (1.263–2.110)****

*n.s.* not significant; ***p = 0.002; ****p < 0.0001. Cox proportional hazards models were adjusted for age, sex, current smoking, use of antihypertensive medication, systolic blood pressure, diabetes mellitus, body mass index, LDL cholesterol and HDL cholesterol.

**Table 5:** Population quartile of AMA in relation to incident heart failure and incident atrial fibrillation.

	Q1	Q2	Q3	Q4
**Incident heart failure**				
	(n = 1219)	(n = 1221)	(n = 1220)	(n = 1119)
Number of events	64	57	65	92
Hazard ratio (95% CI)	(ref)	0.913 (0.638–1.306)^n.s.^	1.008 (0.713–1.125)^n.s.^	1.428 (1.035–1.970)**
**Incident atrial fibrillation**				
	(n = 1190)	(n = 1184)	(n = 1160)	(n = 1141)
Number of events	135	153	148	197
Hazard ratio (95% CI)	(ref)	1.151 (0.912–1.452)^n.s.^	1.133 (0.896–1.433)^n.s.^	1.556 (1.247–1.943)****

*n.s.* not significant; **p = 0.03; ****p < 0.0001. Cox proportional hazards models were adjusted for age, sex, current smoking, use of antihypertensive medication, systolic blood pressure, diabetes mellitus, body mass index, LDL cholesterol and HDL cholesterol.

**Figure 4:**
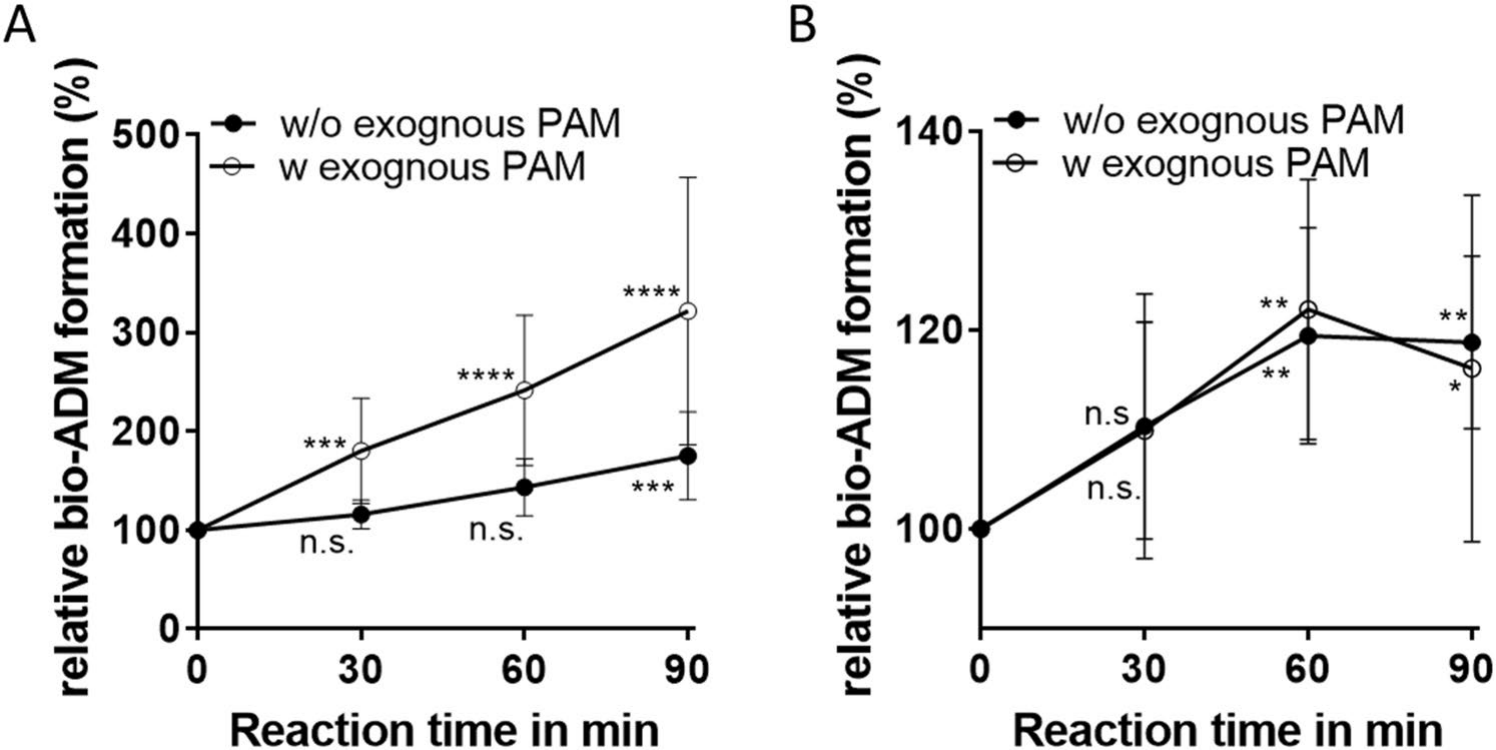
Conversion of endogenous ADM-Gly to bio-ADM in absence (closed circles) and presence (open circles) of exogenous PAM in Li-heparin samples from critically ill subjects (**A**) or from self-reported healthy specimen (**B**). Each point represents means from n = 8 individual reactions. The generated bio-ADM concentrations determined in each sample were normalized to bio-ADM levels at t = 0 min that were set as 100%. Difference in bio-ADM formation was analysed via two-way ANOVA (Dunn’s correction). *p = 0.02; **p = 0.002–0.004; ***p = 0.0004–0.0007; ****p = 0.0001; *n.s.* not significant.

Using the known concentration of recombinant calibration material, we estimated the concentration of active circulating PAM. The concentrations were in ng/mL range (Median: 419.2 ng/mL; Mean: 428.3 ng/mL, 95 CI: 425–431.5 ng/mL). Since the physiological role of circulating amidating activity remains unclear, we addressed the question whether circulating PAM is capable of conversion of circulating ADM-Gly into its amidated form. To this end, we used plasma samples from healthy (mean ADM-Gly value: 6.86 ± 2.2 pg/mL; n = 8) and from critically ill individuals (mean ADM-Gly value: 102.8 ± 54 pg/mL; n = 8). Upon incubation at 37 °C, time dependent production of bio-ADM was observed in plasma from critically ill patients, without addition of exogenous PAM and ADM-Gly. Formation of bio-ADM and therefore consumption of ADM-Gly was increased when exogenous recombinant PAM was added to plasma of critically ill specimen (Fig. 4A). In contrast, while significant but weak bio-ADM formation was observed in plasma from healthy specimen, no additional effect of exogenous PAM addition was present on the bio-ADM formation rate (Fig. 4B).

### In vivo analyses of PAM

To investigate whether circulating PAM is capable of contributing to ADM-Gly amidation in vivo, an animal study with three treatment-groups (PAM, ascorbate and PAM + ascorbate) and one placebo group was conducted.

**Figure 5:**
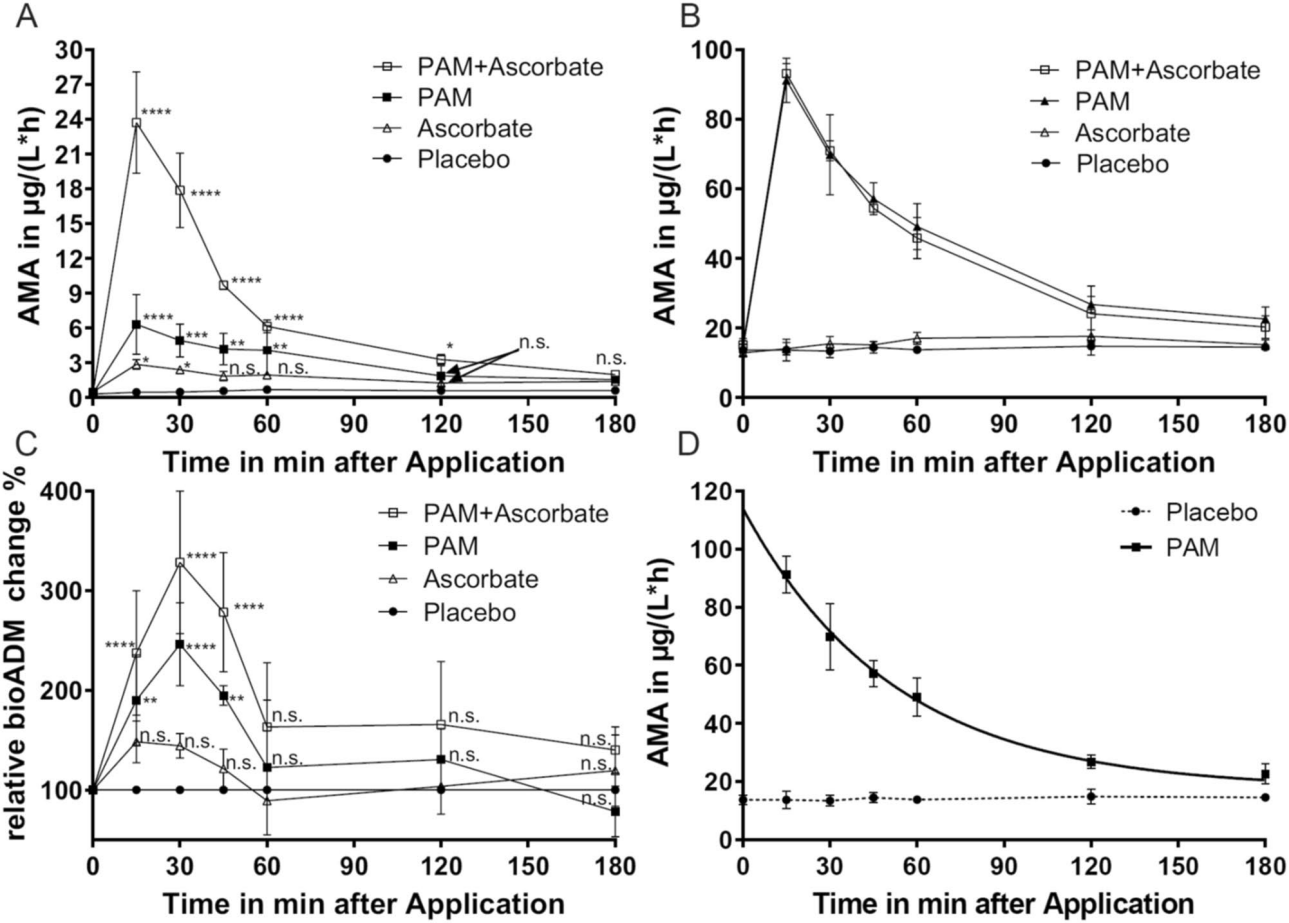
Intravenous injections of placebo (closed circles), PAM (closed squares), Ascorbate (open triangles) and a combination of both (open squares) in n = 3 rats per group. The effect of injected compounds was tested in vitro: (**A**) PAM-AMA assayed in absence of exogenous ascorbate. (**B**) PAM-AMA assayed in presence of exogenous ascorbate. (**C**) Effect of injected compounds on circulating bio-ADM levels. Bio-ADM levels were normalized to levels of Placebo (set as 100%) for each time-point. (**D**) Half-life fit of recombinant PAM in rats, determined from one-phase decay model. Significance of differences was analysed via two-way ANOVA (Dunn’s correction). Significances shown in panels (**A**) and (**C**) are summarized in Table S1.

As shown in Fig. 5A, in all three treatment groups significant elevation of AMA was observed 15 min after injection of respective compounds, when assayed in absence of exogenous ascorbate. In comparison to the placebo group, AMA elevation remained significant for 30, 60 and 120 min in the ascorbate, PAM and PAM + ascorbate groups, respectively (Fig. 5A, Table S1). The observed elevation of AMA was higher in the PAM group than in the ascorbate group. A combined injection of PAM and ascorbate resulted in the highest elevation of AMA (Fig. 5A).

When assayed in presence of exogenous ascorbate, AMA elevation in the PAM- and PAM + ascorbate combination groups was comparable, while in the ascorbate group no effect on AMA was observed in comparison to the control group (Fig. 5B).

To address the question, whether circulating PAM will amidate circulating ADM-Gly in vivo, we analysed changes of plasma bio-ADM levels in all experimental groups (Fig. 5C). As summarized in Table S1, a significant elevation of circulating bio-ADM was observed in the PAM- and PAM + ascorbate treatment groups within of 45 min past injection of the respective compounds (Fig. 5C). Changes of circulating bio-ADM in the ascorbate group were not significant when compared to the placebo group. Consistently with AMA shown in Fig. 5A, circulating bio-ADM elevation was higher in the PAM + ascorbate group than in the PAM group. The short-term effect of PAM injection on circulating bio-ADM levels is consistent with the relatively short half-life of recombinant PAM in circulation. AMA determined in presence of exogenous ascorbate in the PAM group (Fig. 5B) was used to determine the half-life of recombinant PAM in rat, which is 47 min (Fig. 5D).

In combination with the in vitro amidation experiments (Fig. 4), these data show that amidation of circulating ADM-Gly is possible by application of recombinant PAM into circulation. Further, these data show a stimulatory in vivo effect of vitamin C on circulating PAM with respect to ADM-Gly amidation.

## Discussion

State of the art α-amidating assays described in literature mostly use labelled synthetic tripeptides involving chromatographic separation of products and substrates. Especially the usage of radiolabeled substrates, the involvement of chromatographic steps and time-consuming reaction-times might be limiting factors for large scale sample screening. To eliminate those disadvantages, such as substrate and product separation, we developed a robust amidation assay to allow time-efficient large scale sample screening. Additionally, the 96-well based assay format and the absence of chromatographic separation steps gives a solid foundation for a potential automation of the PAM-AMA assay. The amidation reaction leads to linear product formation under described conditions within 1 h. Typically, it was sufficient for the reaction to proceed for 40 min. The reaction mixture can be further transferred directly to the commercially available sphingotest bio-ADM assay^32^ for product quantification without any modifications. In contrast to previously reported assays using artificial substrates, the implementation of non-modified 1–53 ADM-Gly as a substrate reflects the amidation of a naturally occurring full length substrate. Except for substance P-Gly utilizing assays, no comparable amidation assays were described in literature to date^37,38^. To avoid high-dose effects within sphingotest bio-ADM assay during product quantification, the activity assay is limited to substrate concentrations far below the K_M_ values (µM range) reported for alternative substrates^13,14,37^. Substrate variations will therefore influence the overall conversion rate, but since an enzymatic calibrator with pre-defined amidating activities is used, the fitted activities are not influenced by alterations in substrate concentration (Table 1, Fig. S4). A reliable assay performance with serum as an aggressive sample matrix was achieved by preventing ADM degradation due to the use of Amastatin and Leupeptin, combined with an N-terminal anti-ADM antibody (Fig. S2).

The use of an enzymatic calibrator under given assay conditions revealed the active circulating PAM concentration being in ng/mL range. This estimation is limited by the active portion of the used calibrator material and encompasses only the active portion of PAM in circulation. However, our estimation of circulating PAM concentration is in agreement with the mass spectrometric estimation available on the protein atlas database (59 ng/mL; access code: ENSG00000145730). For a reliable determination of circulating PAM concentrations, a sandwich immuno-assay as described for human DPP3 is of high interest^39^.

Using our amidation assay procedure, we determined the distribution of the amidating activity in more than 4900 individuals. To the best of our knowledge this is the first description of serum amidating activity in a large human population-based cohort. Amidating activities were in the range of 3.8 and 72 AMA-units and were Gauss-like distributed. The high activity of 72 AMA-units was found in one single subject within the study population. In consistence with the initial description of amidating activity by Wand and colleagues in a small sample cohort with 7 males and 8 females^13^, no statistically significant sex differences were observed in this work (Fig. S5A). As expected, a significant correlation of PAM-AMA with bio-ADM levels and MR-proADM levels was observed (Fig. S8A,B). MR-proADM is not subject of amidation but it is considered to be a surrogate marker for amidated bioactive adrenomedullin^40^. Although MR-proADM and ADM-Gly arise from the same propeptide, their stoichiometric relationship is imperfect: nascent ADM-Gly is finally amidated, partially and to a varying extent^19,41^. The reduction of AMA in prevalent diabetes and a negative correlation of AMA to the glucose level are in a good agreement with present literature data suggesting impaired Chromogranin A amidation in diabetes, thus reducing insulin granule packaging and secretion^29^. The reduced PAM-AMA in prevalent diabetes might go along with reduced insulin secretion, suggesting pancreatic β-cells as one source of circulating PAM.

We further show a significant elevation of PAM-AMA in subjects with increased SBP and DBP in comparison to normotensive subjects (Fig. 3F–I). Additionally, the highest quartile of AMA showed long-term predictive value with respect to all-cause mortality, cv-mortality, heart failure and atrial fibrillation. A recent study connected a SNP in the PAM allele with an increased risk of hypertension, unfortunately without providing PAM activities in the analysed cohort^42^. Since it was shown lately that PAM contributes to the formation of secretory vesicles in the heart atrium and thereby is involved in atrial natriuretic peptide (ANP) and brain natriuretic peptide (BNP) secretion, elevated PAM-AMA might be associated with increased ANP and/or BNP secretion, both counteracting increased blood pressure due to their natriuretic action^30,43–45^. In consistence, both, ANP and BNP or their midregional or N-terminal propeptides were previously described as predictors of hemodynamic stress including AF^46^, HF^47^ and mortality^48^. On the other hand, bioactive ADM or its midregional surrogate MR-proADM are known to be elevated in patients with hypertension^49–51^, HF^52–55^, and AF^55,56^. Elevation of bio-ADM in several pathological condition is proposed to act protective in cardiovascular homeostasis maintenance^50^. Therefore, increased PAM-AMA within of observations made in this study might reflect increased co-release of PAM during ADM secretion. The relatively weak but significant correlation of AMA with bio-ADM and MR-proADM levels supports this assumption. These associations of PAM in conditions of hemodynamic stress implicate the involvement of PAM being of non-catalytic nature. But since the concentration of PAM as the source of amidating activity is substantial in circulation (ng/mL range), it is intriguing whether circulating PAM contributes to amidation of circulating peptidylglycines, such as ADM-Gly. Alterations of ADM-Gly amidating activity in hypertensive subjects were discussed previously^50^. Similar as stated by Shimosawa et al.^50^ increased PAM-AMA in hypertensives could contribute to an active amidation of circulating ADM-Gly thereby increasing hypotensive bio-ADM in order to reduce blood pressure in hypertension. An elevation of AMA in conditions of hemodynamic stress might result from elevated secretion of amidated peptides and their glycine-extended counterparts or function as an indicator of the elevated need of peptide amidation that is partially compensated by circulating PAM. Contrary, amidation of circulating peptidylglycines by circulating PAM is considered to be unlikely due to insufficient cofactor concentrations in circulation^57^. Since in vitro amidation is possible without exogenous ascorbate^13,14,16^ (Fig. 5A) and, residual test-tube activity of human body-fluid PAM without exogenous copper addition was shown^13,14^, the possibility of amidation in circulation should not be overlooked with respect to low circulating copper and ascorbate concentrations. Cao et al. were able to show ADM-Gly amidation by isolated rat aorta, without addition of either copper or ascorbate^58^ and the artificial elevation of circulating PAM-AMA in rats (this work, Fig. 5A,B) resulted in elevation of circulating bio-ADM in vivo (Fig. 5C). Therefore, the increased PAM-AMA in hemodynamic stress could contribute to active amidation of circulating peptidylglycines, such as ADM-Gly. Our in vitro amidation experiments with plasma from healthy and severely ill individuals show that in vitro amidation of circulating ADM-Gly by circulating PAM is possible. The minor bio-ADM generation from plasma of healthy subjects by circulating PAM without exogenous substrate addition in this work disagrees with the work of Kitamura et al., stating ADM-Gly as the major circulating form of ADM^19^. Our in-house ADM-Gly determination in plasma from healthy humans (6.86 ± 2.2 pg/mL) shows low concentrations of circulating ADM-Gly in healthy individuals and explains the weak bio-ADM elevation by endogenous PAM (Fig. 4B). In contrast, when using Li-Heparin plasma from critically ill subjects, bio-ADM synthesis by endogenous PAM was possible in a time dependent manner and bio-ADM production rate was increased after exogenous PAM addition (Fig. 4A). Even though these amidation experiments were conducted in presence of optimal cofactor concentrations, they show the potential of ADM-Gly amidation by PAM in circulation. The role of circulating PAM in amidation of circulating peptidylglycines is supported by the short-term bio-ADM increase in rats after PAM injection (Fig. 5C). The short-term nature of bio-ADM elevation by recombinant PAM in circulation might be attributed to the relatively short half-life of recombinant PAM (47 min) in circulation (Fig. 5D). To further investigate the active amidation in circulation, determination of circulating peptidylglycines levels in combination with their amidated counterparts and circulating PAM-AMA is of high interest. Additionally, a correlation of serum AMA to ANP or MR-proANP levels could provide deeper insight into the involvement of PAM in natriuretic peptide secretion.

We further observed a stimulatory in vivo effect of ascorbate-injection on circulating PAM-AMA in rats. The subsequent AMA elevation indicates that concentrations of circulating reducing agents do not stimulate total circulating PAM and Ascorbate application is suitable for this purpose. Unexpectedly, the stimulatory effect of ascorbate on recombinant PAM was higher when compared to endogenous rat enzyme (Fig. 5A). This effect cannot be explained with the present dataset and requires further investigation. The role of ascorbate on peptide amidation was previously studied in animal models. Deprivation of ascorbate resulted in a decrease of amidated gastrin or melanocyte stimulating hormone (α-MSH) and an increase of their glycine-extended counterparts^20,21^. Centrally administered ascorbate on the other hand elevated levels of circulating vasopressin in rats^22^. In humans, it was speculated that depletion of ascorbate in sepsis might contribute to the observed decrease in vasopressin biosynthesis^25,59^. Ascorbate as a cofactor for PAM was further discussed in connection of beneficial effects of vitamin C administration and sepsis outcome^23–26^. In corresponding studies high dose ascorbate was administered intravenously, thus one could speculate that this would lead to an overall increased PAM activity in circulation. Our data support this hypothesis: On the one hand oral vitamin C uptake in healthy human volunteers had a stimulatory effect on circulating PAM-AMA, when assayed in absence of exogenous ascorbate (Fig. S10), on the other hand ascorbate injection in rats in this work stimulated endogenous PAM (Fig. 5A) and when co-injected with recombinant PAM led to significant elevation of circulating bio-ADM levels (Fig. 5C, Table S1). In these animal experiments bio-ADM was analysed as a model peptide undergoing amidation by PAM and no significant elevation of bio-ADM was observed after ascorbate injection. Little is known about substrate preferences of PAM, thus it is feasible that amidation of other glycine-extended PAM substrates is preferred by circulating PAM.

In summary, this report describes a broad distribution of amidating activity (AMA) in a large human sample cohort. We found a predictive value of serum AMA with respect to mortality, incident heart failure and incident atrial fibrillation. Further, elevated AMA correlates with untreated systolic and diastolic hypertension. In addition, we show that ascorbate may be used as a stimulator of circulating PAM and we provide experimental evidence for amidation of circulating ADM-Gly by circulating PAM. To the best of our knowledge, this is the first direct experimental link of circulating PAM and peptide amidation. Our understanding of the presence of glycine-extended PAM substrates in circulation is rather limited and no human disease condition has been directly attributed to PAM or it’s insufficiency, yet. A stronger future focus and research interest is necessary for the connection of the PAM enzyme to disease states that could be associated with impaired amidation status of peptide hormones. A characterization of amidated and glycine-extended forms of peptide-hormones in connection with amidating activity in healthy and diseased subjects would provide a deeper insight into the use of PAM as a biomarker. Given the capability of PAM to amidate circulating peptidylglycines, it might be a promising therapeutic target.

## Materials and methods

### ADM measurements

Quantification of bio-ADM was conducted using the sphingotest bio-ADM assay as described elsewhere^32^. In order to quantify ADM-Gly, the sphingotest bio-ADM assay was modified as follows: the tracer material was replaced by an ADM-Gly specific MACN labelled antibody (AK835/G4), that does not react with bio-ADM. The ADM-Gly specific assay was calibrated with synthetic human ADM-Gly (custom synthesis by Peptides&Elephants, Hennigsdorf, Germany). The MACN labelling procedure was previously described^32^.

### Recombinant PAM calibrator material

Recombinant PAM was purchased from SinoBiological (cat-no.: 13624-H08H). A 6-point calibrator was prepared by dilution of recombinant PAM in 100 mM Tris–HCl pH 7.5, 2.5% BSA to the final concentration of 4000 ng/mL, 2000 ng/mL, 666.7 ng/mL, 222.2 ng/mL, 74 ng/mL and 0 ng/mL. Internal assay controls were undiluted and threefold diluted human serum. Amidating activity (AMA) of calibrators and controls was defined by subjecting the samples to an amidation assay: calibrators (20 µL) or controls (20 µL) were pipetted into 96 well polypropylene plates prefilled with 20 µL of 100 mM Tris–HCl, pH 7.5. Subsequently, 160 µL of reaction buffer (RB) (100 mM Tris–HCl, 6.25 µM CuSO4, 2.5 mM l-ascorbate, 125 µg/mL bovine liver Catalase, 62.5 µM Amastatin, 125 µM Leupeptin, 375 µg/mL N-terminal anti-ADM antibody (HAM1101^32^), 36 ng/mL synthetic human 1–53 ADM-Gly) were added. To generate a time-point 0 min, 100 µL of each individual reaction were inactivated with 20 mM EDTA. All reactions were incubated without aspiration at 37 °C for 40 min. Finally, the remaining samples were inactivated with 20 mM EDTA and subsequently subjected to the sphingotest bio-ADM assay^32^. The concentration of generated bio-ADM was used to define the AMA of each calibrator-point (Eq. 1).

1 $$\frac{{\left( {[bioADM]\frac{pg}{{mL}}^{t40} - [bioADM]\frac{pg}{{mL}}^{t0} } \right)}}{40\;\min } \times 60\min \times DF$$

with DF—dilution factor.

The resulting activity is measured in AMA-units, where 1 AMA-Unit is defined as 1 µg bio-ADM generated in 1 L of sample in 1 h. Each calibrator- and control sample were analysed in duplicate in 4 independent experiments. The typical calibrator-activity was in the range of 0.7 and 122 AMA-units.

### Amidating activity measurements

#### Amidating activity with enzymatic calibrator

The AMA of sample PAM was determined in a soluble assay as described in “Recombinant PAM calibrator material” section. The bio-ADM derived signals (relative light units (RLU)) were used to calculate unknown AMA from the samples. The RLU generated at t = 0 min were subtracted from RLU generated at t = 40 min. The ΔRLU (t40 − t0) of the calibrator material with pre-defined AMA were used to determine the PAM-AMA in sample material using the cubic-spline fit algorithm of GraphPad Prism Version 7.0. Estimation of circulating PAM concentration was achieved by using ΔRLU (t40 − t0) of sample material and fitting versus ΔRLU (t40 − t0) of the calibrator material of known PAM concentration. Each sample was analysed as duplicates. Samples and calibrators were treated equally during the whole procedure.

#### Product-formation linearity testing

Human serum was diluted 1-, 2-, 4-, 8-, 20- or 40-fold in 100 mM Tris–HCl, pH 7.5 in a total volume of 200 µL. Tris–HCl, pH 7.5 was used as a control. The reaction was started by addition of 800 µL of RB followed by incubation at 37 °C without aspiration. After 0, 5, 10, 15, 20, 25, 30, 40, 50 and 60 min, 100 µL of each reaction mixture were inactivated with 20 mM EDTA. Bio-ADM was quantified using the sphingotest bio-ADM assay^32^.

#### Bio-ADM formation in absence of exogenous ascorbate

60 µL of sample matrix were spiked with 20 µL of ascorbate-free reaction buffer (RB-AF): (100 mM Tris–HCl, pH 7.5, 200 µM Amastatin, 800 µM Leupeptin, 400 µg/mL bovine-liver catalase, 20 µM CoSO4, 1.2 mg/mL N-terminal anti-ADM antibody (HAM1101^32^) and 345 ng/mL of synthetic human 1–53 ADM-Gly). Subsequently, 40 µL of each reaction were inactivated with 20 mM EDTA. All reactions were incubated for 60 min at 37 °C and inactivated with 20 mM EDTA. Afterwards each sample was diluted twofold with 100 mM Tris–HCl, pH 7.5 and quantified for bio-ADM with the sphingotest bio-ADM assay^32^.

#### Bio-ADM formation from endogenous ADM-Gly by endogenous PAM

360 µL of Li-Heparin were spiked with 90 µL of substrate-free reaction buffer (RB-SF): [100 mM Tris–HCl, pH 7.5, 10 mM l-ascorbate, 250 µM Amastatin, 1 mM Leupeptin, 500 µg/mL bovine-liver catalase, 25 µM CoSO4 and 1.5 mg/mL N-terminal anti-ADM antibody (HAM1101^32^)] in presence or absence of recombinant human PAM (500 ng/mL). The reactions were incubated at 37 °C and inactivated after 0, 30, 60 and 90 min with 20 mM EDTA. Produced bio-ADM was quantified with the sphingotest bio-ADM assay^32^.

### Assay performance studies

The inter-assay CV was determined by screening of ten plasma samples (range 1.59–88.4 AMA-units) by two operators on 10 days in a total of 16 independent assays. Each screening run was analysed in a separate sphingotest bio-ADM assay^32^. The limit of quantification (LOQ; functional assay sensitivity) was defined as the PAM-AMA quantifiable with a CV of 20%. The limit of detection (LOD) was determined as described by Armbruster et al.^60^.

The intra-assay CV was determined by activity measurement of three serum samples with varying activity. Activity of each sample was determined as 32-times iteration within of a single assay.

Accuracy was assessed by spiking of serum with recombinant PAM (SinoBiological) of known concentration (range 0–2750 ng/mL). The determined PAM-AMA levels in spiked serum samples was compared to expected activities. Expected activities were calculated form determined Serum-AMA and determined recombinant PAM-AMA. The testing was performed with serum samples from three self-reported healthy volunteers in three independent experiments.

For linearity studies, three random serum samples (low-pool) were diluted stepwise by 10% (range 1:10–1:100 in assay) with zero-matrix (100 mM Tris, 2.5% BSA, pH 7.5). Further, serum spiked with 3.1 µg/mL of recombinant PAM (high-pool) was diluted stepwise by 10% in untreated serum (low-pool), and the deviation of measured PAM-AMA from expected concentrations and activities was calculated.

Mixing recovery was assessed by using twelve random serum samples with varying, predetermined PAM-AMA. Pools (n = 12) were generated by 1:1 combination of two different serum samples. PAM-AMA deviation from the expected value was determined.

Variation of substrate concentration was performed by using 10, 20, 28.8 and 40 ng/mL of 1–53 ADM-Gly in the activity assay calibrated with recombinant human PAM.

The acceptance criterion for all performance studies was ± 20% difference from the original or expected PAM-AMA.

### Stability testing

#### Stability of amidating activity in human serum

Serum aliquots from three independent donors were frozen at − 80 °C. Every 24 h single aliquots were thawed and subjected to storage either at 37 °C, room temperature or 4 °C. Freeze–thaw stability was assessed by slow thawing of aliquots every 24 h with vortexing for 10 s and re-freezing at − 80 °C. Activity in control samples that did not undergo several freeze–thaw cycles or storage at different temperatures was set to have 100% amidating activity.

#### Serum-stability of adrenomedullin

Human serum was 1:5 diluted with reaction buffer (RB), RB devoid of Amastatin and Leupeptin (RB-AL) or RB-AL devoid of the N-terminal anti-ADM antibody (HAM1101^32^) (RB-0). The used RB, RB-AL and RB-0 contained either ADM-Gly (28.8 ng/mL) or equal concentration of bio-ADM. Final concentrations of all RB components were as described in “Amidating activity (AMA) with enzymatic calibrator” section. Prepared samples were incubated at 37 °C for 120 min. After 0, 30, 60, 90 and 120 min, aliquots (300 µL) were removed from the incubation mixtures and immediately frozen and stored in liquid nitrogen. Remaining ADM-Gly or bio-ADM were quantified as described in “ADM measurements” section.

### MPP study population

To assess the normal distribution of PAM-AMA in a broad population, serum samples from the Malmö Preventive Project (MPP) were measured. The Swedish single-centre, prospective, population- based study is described elsewhere^61^. Of the 18,240-participant study, a randomly selected subcohort was used (n = 4942) to determine the normal range of PAM-AMA in serum. The methods used for assessment of baseline cardiovascular risk factors^62^, as well as record linkage with the Swedish national inpatient and cause of death registries used for endpoint retrieval of mortality, cardiovascular mortality, atrial fibrillation and congestive heart failure are described in detail previously^55,62,63^.

All participants signed a written informed consent form before entering MPP-RES. The study was approved by The Regional Ethical Review board at Lund University, Sweden (LU 244-02) and complied with the Helsinki Declaration.

### In vivo and animal analyses

Endotoxin-free, recombinant PAM (SinoBiological) was buffer exchanged into sterile phosphate buffer saline (PBS, Dulbecco) and adjusted to 50 µg/mL. Sterile ascorbate solution for injection (200 mg/mL, vitamin C 1000, WÖRWAG Pharma) was purchased in a local pharmacy and adjusted under sterile conditions with PBS to 40 mg/mL. For combined injections of PAM and ascorbate, the compounds were prepared separately in equal volumes (100 µg/mL or 80 mg/mL for PAM and ascorbate, respectively). Both compounds were combined directly prior to injection to result in concentrations of 50 µg/mL for PAM and 40 mg/mL for ascorbate. Sterile PBS was used as placebo. All samples were stored at − 80 °C until use. Animals, male Wistar rats (Charles River Labs, Sulzfeld) 8 weeks of age at arrival, were assigned randomly (no allocation parameter) into 4 groups (placebo, ascorbate, PAM, PAM + ascorbate) with n = 3 animals per group. Animals were allowed to access tap water and food ad libitum, were kept under 12 h/12 h light–dark cycle conditions and were granted 8 days of acclimatization following arrival. Each animal in the individual group received 500 µL of the respective compound intravenously (lateral tail vein) as a single dose injection. Blood sampling was performed from each animal in each group as Li-Heparin 30 min prior to- and 15, 30, 45, 60, 120 and 180 min post injection. Approximately 300 µL of blood were drawn per time-point and animal into CB 300 Li-Heparin microvettes (Sarstedt #16.443). Animals were anesthetized with 5 vol% Isoflurane inhalations (IsoVet 1000 mg/g; Dachera, Aulendorf, Germany) prior to injections and blood drawing. Animals were allowed to wake up from anesthesia between the different blood sampling time points. The animal study was conducted by preclinics GmbH, Potsdam, Germany in accordance with the German federal Law for care and use of laboratory animals (Tierschutzgesetz Bundesrepublik Deutschland). The use of animals in this study was registered at the “Landesamt für Arbeitsschutz, Verbraucherschutz und Gesundheit (LAVG)” department V2 of the federal state Brandenburg, Germany. The study protocol was approved by the ethical commission of LAVG. The study is in compliance with the Animal Research: Reporting of In Vivo Experiments (ARRIVE) guidelines. All animals were housed and handled according to guidelines from the Federation of Laboratory Animal Science Associations (FELASA).

### Ascorbate effect on circulating human PAM activity

Healthy volunteers (n = 4) received 2000 mg of vitamin C (Dr. Scheffler, Additiva Vitamin C) as an oral single dose. Blood sampling was performed prior to- and 1, 2 and 3 h post administration. Amidating activity was determined from Li Heparin plasma and serum.

### Patients and controls

Anonymized human serum or Li-heparin plasma samples from healthy individuals were either purchased from InVent Diagnostica GmbH, Hennigsdorf or were in-house collected from anonymized self-reported healthy volunteers.

Samples from critically ill subjects (sepsis or septic shock) were purchased from InVent Diagnostica GmbH, Hennigsdorf.

### Statistical analysis

Statistical analyses were performed with GraphPad Prism 7.0 and SPSS version 26. Distribution was tested with the D'Agostino–Pearson omnibus test for normal distribution. Comparison of nonparametric data was done with the Mann–Whitney *U* test. Multiple comparisons were performed using one-way ANOVA or two-way ANOVA as indicated. Correlations were calculated as Spearman rank correlation. P values < 0.05 were considered significant. Linearities were tested using Runs test. Baseline levels of AMA in the MPP (expressed as quartiles, with quartile 1, i.e. with lowest values of AMA, defined as the reference quartile) were related to endpoints during follow-up (incident heart failure, incident atrial fibrillation, cardiovascular mortality and all-cause mortality) using Cox Proportional Hazards Model adjusted for baseline risk factors (age, sex, current smoking, use of antihypertensive medication, systolic blood pressure, diabetes mellitus, body mass index, LDL cholesterol and HDL cholesterol).

## Supplementary Information

Supplementary Information.

## Abbreviations

ADM: Total adrenomedullin irrespective of amidation status
ADM-Gly: C-terminally glycine extended adrenomedullin
AF: Atrial fibrillation
AMA: Amidating activity
ANP: Atrial natriuretic peptide
bio-ADM: C-terminally amidated bioactive adrenomedullin
BNP: Brain natriuretic peptide
CCK: Cholecystokinin
DBP: Diastolic blood pressure
DPP3: Dipeptidyl peptidase 3
HDL: High density lipoprotein
HF: Heart failure
ILMA: Immunoluminometric assay
LOD: Limit of detection
LOQ: Limit of quantification
MR-proADM: Mid-regional proADM
MR-proANP: Mid-regional proANP
MPP: Malmoe Preventive Project
MSH: Melanocyte stimulating hormone
PAL: Peptidyl-α-hydroxyglycine α-amidating lyase
PAM: Peptidylglycine α-amidating monooxygenase
PHM: Peptidylglycine α-hydroxylating monooxygenase
SBP: Systolic blood pressure
RT: Room temperature
