# Supplementary material for: Novel insights into peptide amidation and amidating activity in the human circulation
Source: Sci Rep. 2021 Aug 4;11:15791. doi: 10.1038/s41598-021-95305-y (PMC8338962; doi:10.1038/s41598-021-95305-y)
Supplement: Supplementary file 1 — Supplementary Information. [file 41598_2021_95305_MOESM1_ESM.pdf]

Supplementary Information for the Scientific Reports Manuscript:

**“Novel Insights into Peptide Amidation and Amidating Activity in the human circulation”**

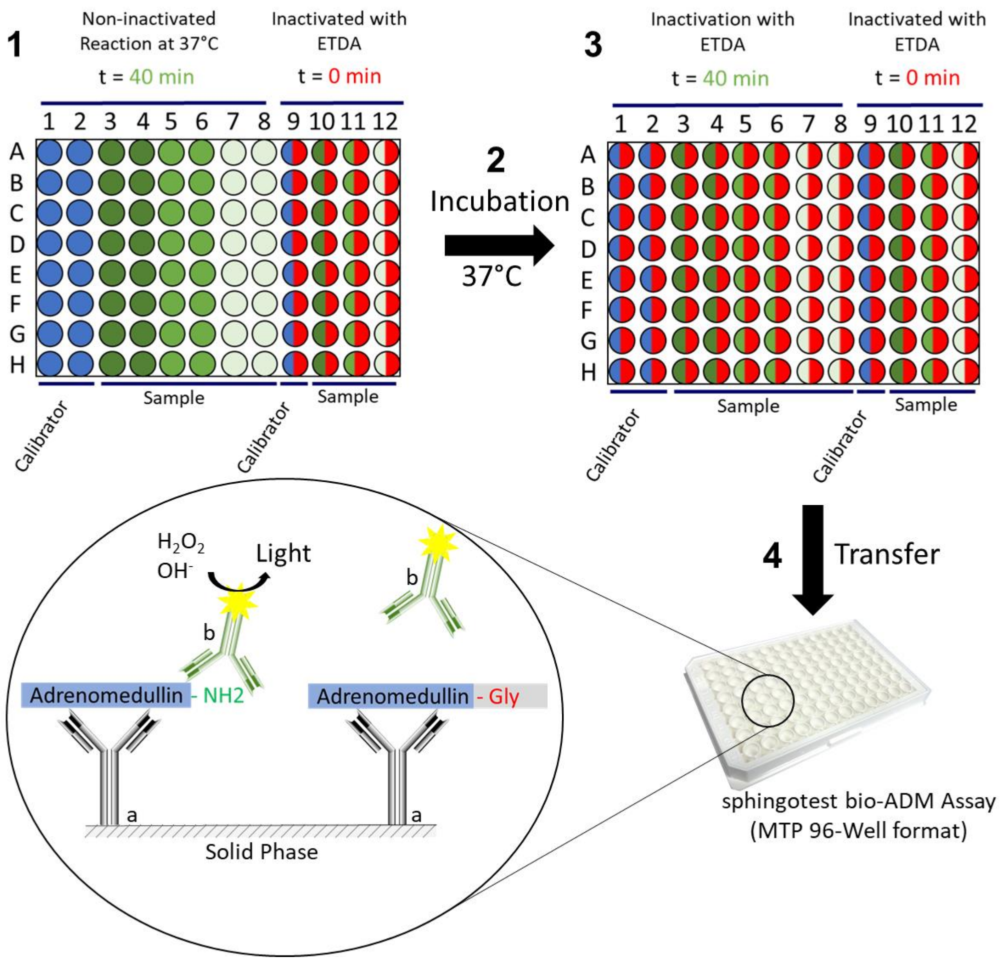


**Supplemental Figure S1:** Schematic overview on the PAM-AMA Assay. In step 1 samples and calibrators are transferred in duplicates (for t=40 min) in a 96-well non-binding PP reaction-plate and an inactivation with EDTA (for t=0 min) is performed as single determination. In Step 2 the reaction plate is incubated at 37°C for 40 minutes. Afterwards inactivation of active sample-material is performed with EDTA (step 3). In step 4 samples are transferred to the sphingotest bio-ADM assay for product quantification: Both, bio-ADM and ADM-Gly are bound by the solid-phase capture antibody (a). The MACN labelled tracer antibody (b) specifically reacts with c-terminally amidated ADM but not with ADM-Gly. For details see the materials and methods section. The figure was created with Microsoft PowerPoint 2016, version 2105.

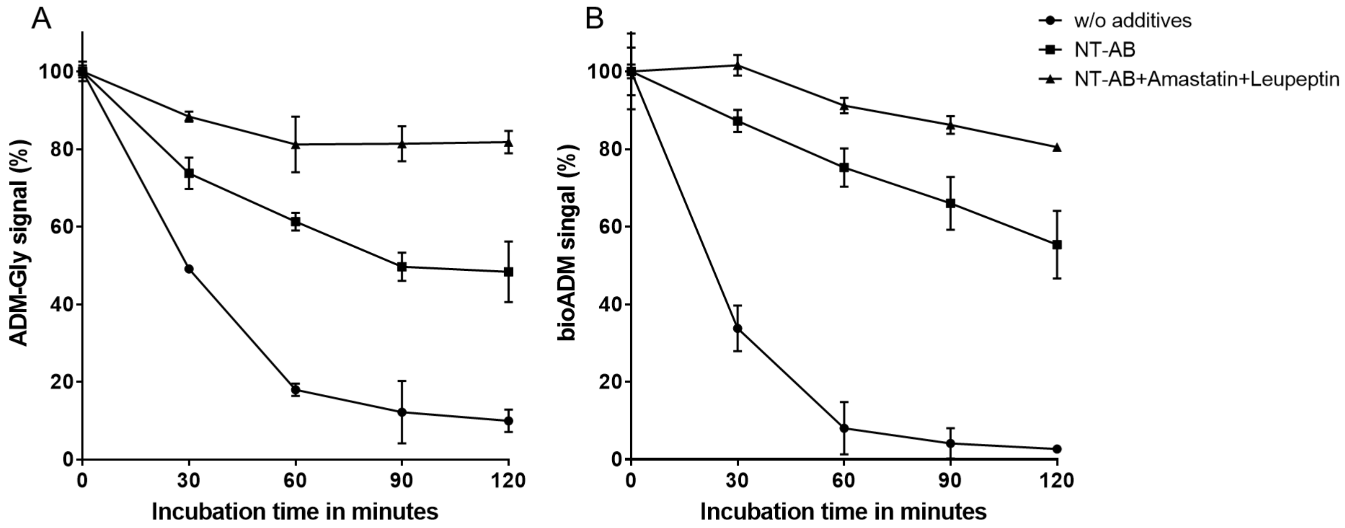


**Supplemental Figure S2**. Stability of synthetic human 1-53 ADM-Gly **(A)** and synthetic human bioactive ADM (bio-ADM) **(B)** under assay-like conditions in presence and absence of stabilizing agents. NT-AB: N-terminal binding Antibody

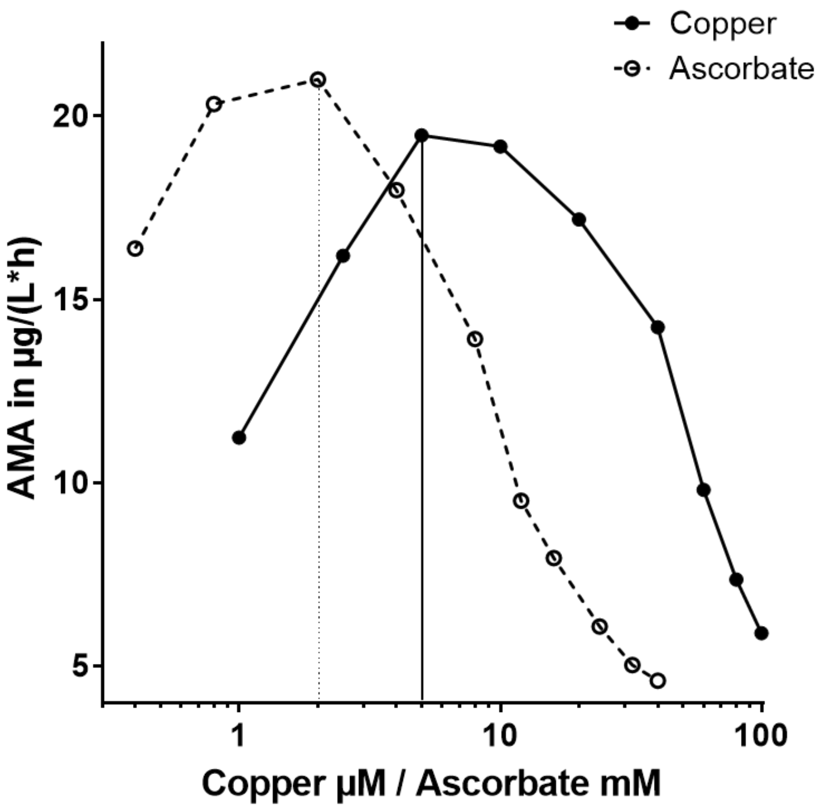


**Supplemental Figure S3. Determination of optimal copper and ascorbate concentrations for human serum PAM.PAM-AMA was measured in a presence of 5 µM CuSO4 and varying ascorbate concentration (open circle). In the following step, the PAM-AMA was determined in the presence of 2 mM ascorbate and variable CuSO4 concentrations (closed circles).**

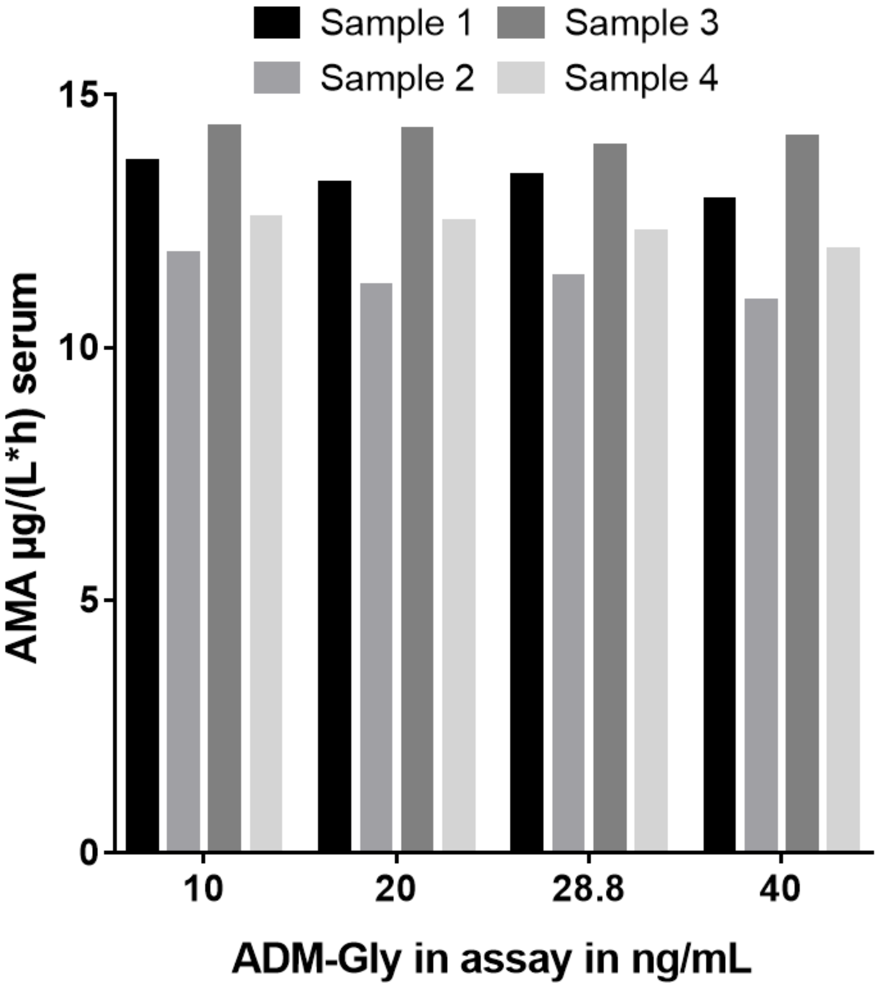


**Supplemental Figure S4.** AMA from 4 independent samples fitted via the recombinant calibrator using varying substrate concentrations

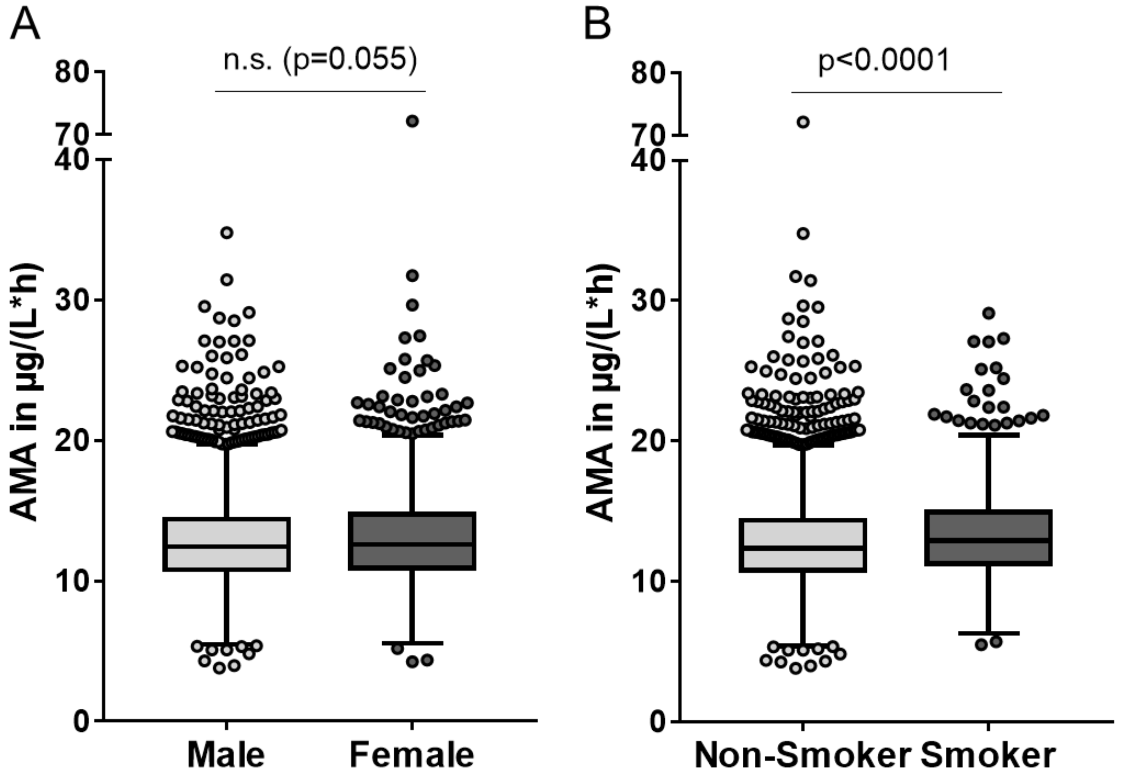


**Supplemental Figure S5**. AMA in the MPP study cohort. Comparison between females (n=3394) and males (n=1548) **(A)** as wells between non-smokers (n=3995) and smokers (n=947) **(B)**. Significancy was tested with non-parametric Mann-Whitney test. n.s: not significant

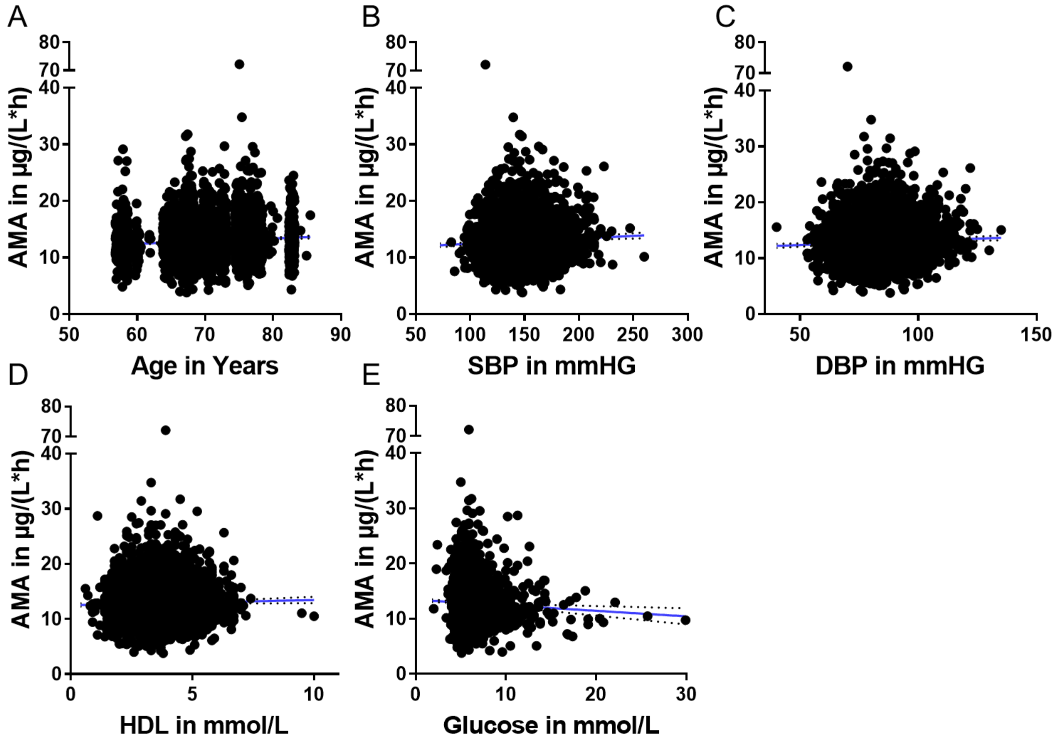


**Supplemental Figure S6**. Spearman rank correlations of PAM-AMA with clinical baseline parameters in the MPP-study cohort. Correlations of Age **(A)**, SBP **(B)**, DBP **(C)**, HDL **(D)** and Glucose **(E)** vs AMA. Number of pairs, correlation coefficients and significancy are summarized in table 3.

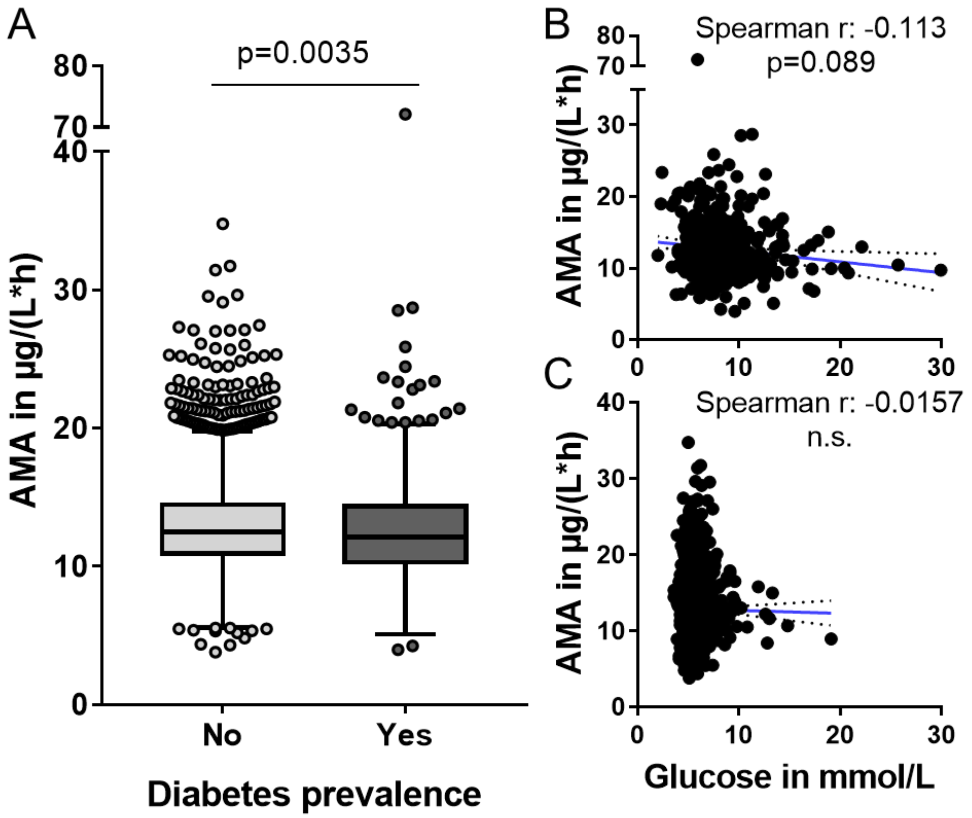


**Supplemental Figure S7**. AMA in the MPP study cohort in subjects with (n=533) and without (n= 4409) prevalent diabetes (A) and Spearman rank correlations of PAM-AMA vs. Glucose in subjects with prevalent Diabetes (B) and in subjects free of Diabetes (C). Significancy in (A) was tested with non-parametric Mann-Whitney test. Number of pairs, correlation coefficients and significancy for (B) and (C) are summarized in table 3.

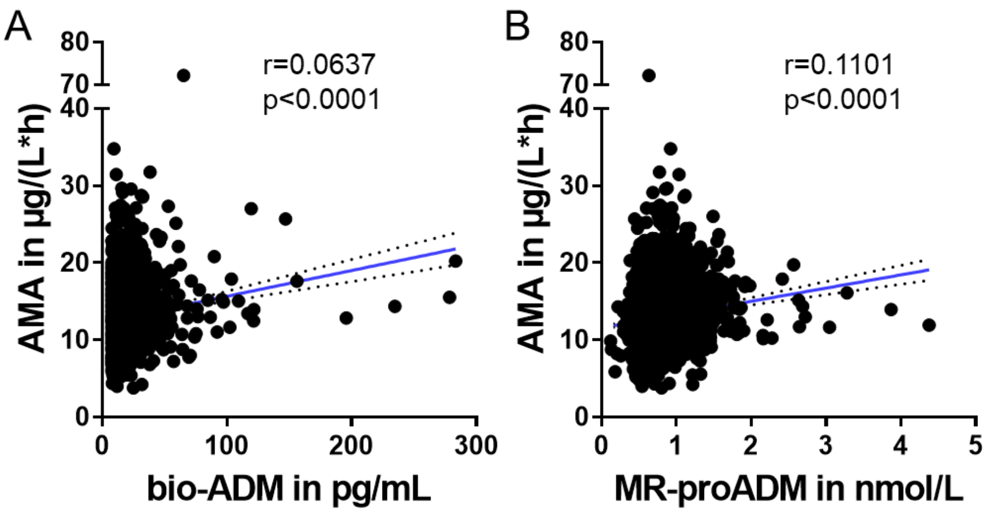


**Supplemental Figure S8**. Spearman rank correlations of PAM-AMA with bio-ADM levels (n=4620 pairs) (A) and MR-proADM levels (n=4942 pairs) (B).

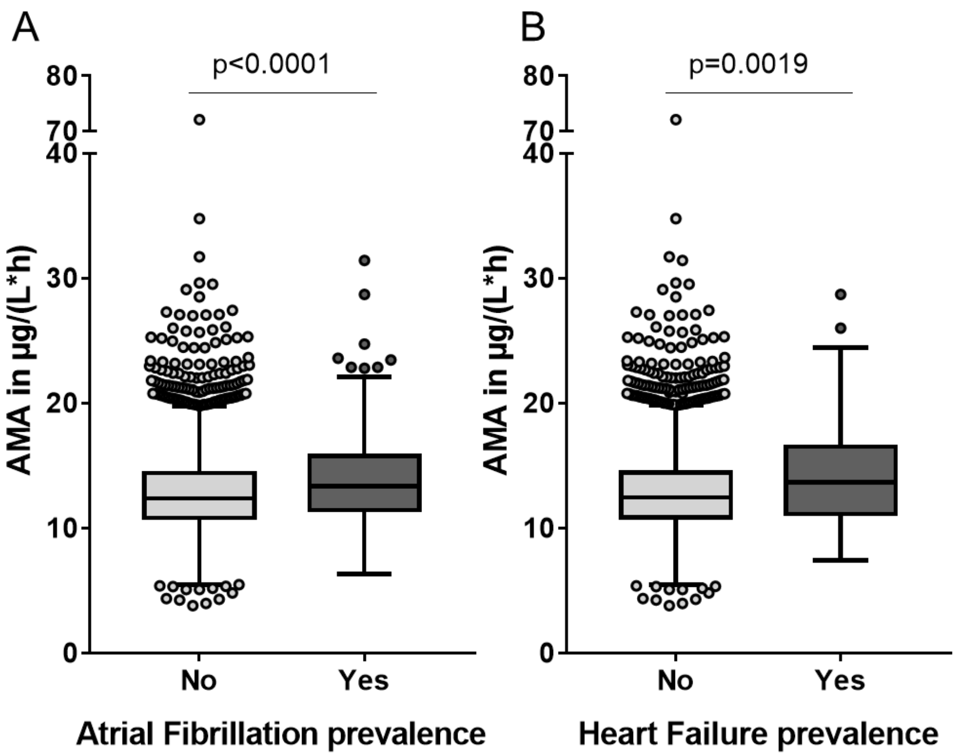


**Supplemental Figure S9**. AMA in the MPP study cohort. Comparison subjects free of atrial fibrillation (n=4675) and with prevalent atrial fibrillation (n=267) **(A),** as wells between subjects free of heart failure (n=4859) and with prevalent heart failure (n=83) **(B)**. Significancy was tested with non-parametric Mann-Whitney test.

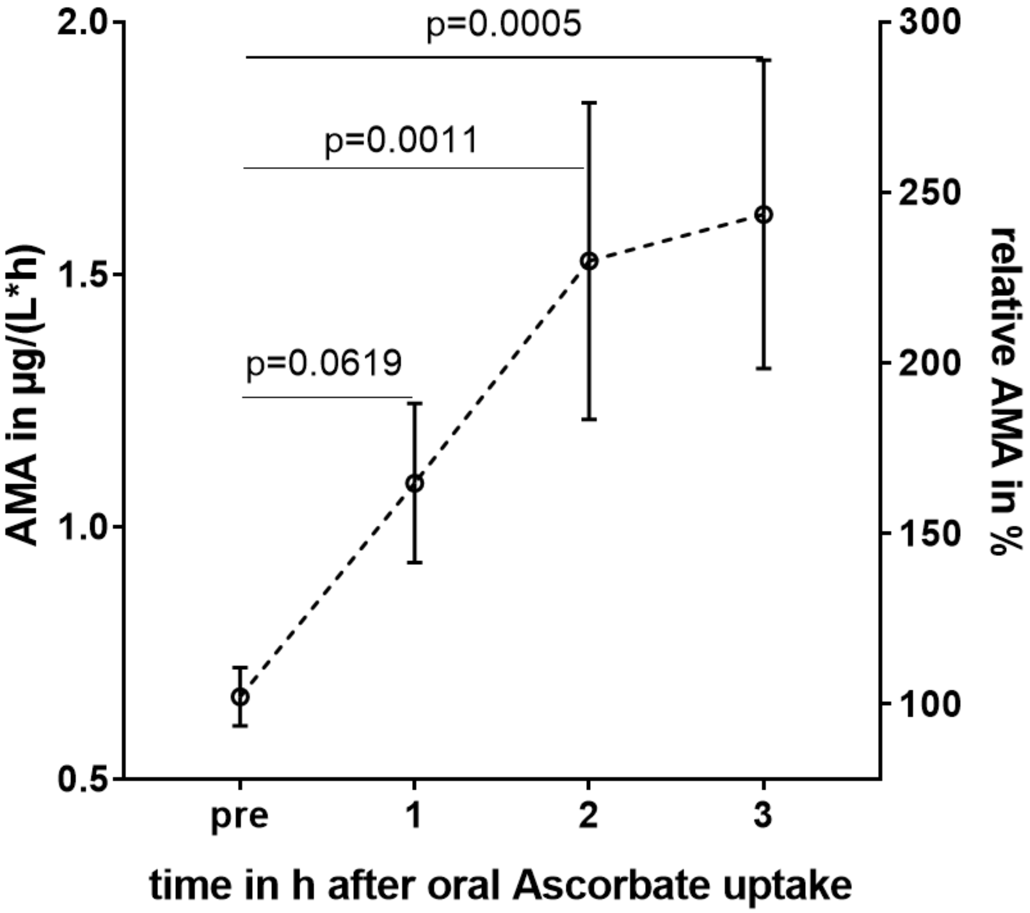


**Supplemental Figure S10**. Amidating activity in healthy human volunteers (n=4) before (0h) and after oral intake of 2000mg of ascorbate, measured without addition of exogenous ascorbate. AMA at t = 0h was set as 100%. Significancy was tested via two-way ANOVA (Dunn's correction).

**Supplemental Table S1:** Two-way ANOVA analysis of data shown in Fig. 7A and 7C (Dunn's correction)

| | Figure 7 A | | | Figure 7 C | | |
|---|---|---|---|---|---|---|
| | PAM | Ascorbate | PAM+Ascorbate | PAM | Ascorbate | PAM+Ascorbate |
| timepoint | p-value | | | p-value | | |
| 0 min | n.s. | n.s. | n.s. | n.s. | n.s. | n.s. |
| 15 min | 0.0001 | 0.0310 | 0.0001 | 0.0059 | n.s. | 0.0001 |
| 30 min | 0.0003 | 0.0472 | 0.0001 | 0.0001 | n.s. | 0.0001 |
| 45 min | 0.0041 | n.s. | 0.0001 | 0.0036 | n.s. | 0.0001 |
| 60 min | 0.0073 | n.s. | 0.0001 | n.s. | n.s. | n.s. (0.0733) |
| 120 min | n.s. | n.s. | 0.0420 | n.s. | n.s. | n.s. |
| 180 min | n.s. | n.s. | n.s. | n.s. | n.s. | n.s. |

**n.s.: not significant**
